# The dual-port endoscope-assisted cyst enucleation on the maxillofacial region

**DOI:** 10.1186/s40902-021-00327-1

**Published:** 2021-10-22

**Authors:** Hyuk Choi, Gyu-Jang Cho, Ki-Hyun Jung, Jae-Yun Jeon, Seung-Weon Lim, Chang-Joo Park, Kyung-Gyun Hwang

**Affiliations:** 1 Department of Dentistry/Oral and Maxillofacial Surgery, College of Medicine, Hanyang University, 222 Wangshimri-Ro, Seongdong-Ku, Seoul, 04763 South Korea; 2 Department of Dentistry/Orthodontics, College of Medicine, Hanyang University, Seoul, South Korea

**Keywords:** Endoscope, Minimally invasive surgery, Jaw cysts

## Abstract

**Background:**

Endoscope-assisted surgery is a surgical method that has been used in oral and maxillofacial surgical fields. It provides good illumination, clear, and magnified visualization of the operative field. The purpose of this article is to describe the early clinical experiences to conduct minimally invasive surgery with endoscope-assisted enucleation of cysts on the jaw. It appears that this approach may be a superior alternative to the conventional approach.

**Methods:**

In this study, 24 patients (9 females, 15 males, average age 41.5) underwent endoscope-assisted cyst enucleation under general anesthesia. All operations were done by one surgeon. The cases were classified depending on whether bone penetration occurred at the cyst site. The cystic lesions were enucleated using an endoscope with a 0°, 1.9 mm diameter, or a 30°, 2.7 mm diameter. Two bony windows were used for the insertion of a syringe for irrigation, curettes, suction tips, sinus blades, surgical drills, and an endoscope. An additional small channel was made for the insertion of endoscopic instruments.

**Results:**

The 24 patients who underwent cyst enucleation were regularly observed for 3 to 12 months to evaluate for complications. Although some patients experienced swelling and numbness, these symptoms did not persist, and the patients soon returned to normal and there was no sign of recurrence.

**Conclusions:**

The results of this study have suggested the possibility of minimally invasive surgery with endoscopes when it comes to cyst removal in the oral and maxillofacial region. Nevertheless, this study has limitations designed as a preliminary report focusing on the feasibility of endoscope-assisted cyst enucleation in the oral and maxillofacial regions.

## Backgrounds

Benign jaw cysts often occur in the oral and maxillofacial areas. Traditionally, cysts of this type are categorized into those with developmental origins and those with inflammatory origins. A typical developmental cyst is odontogenic cyst and odontogenic keratocyst, whereas an inflammatory cyst is a radicular cyst, which occurs most commonly in the oral and maxillofacial area [1]. The most common treatment method is enucleation of the entire cystic region.

If the cystic lesion is large or surrounds a significant anatomic structure, marsupialization is recommended as an alternative treatment. Even though conventional methods of surgical enucleation provide a clear view of the surgical field and reduce procedure time, a wide bony window is required for adequate visibility with the unaided eye, and access to surgical instruments is required. Thus, this procedure frequently causes excessive postoperative pain, bleeding, and swelling.

The concept of minimally invasive surgery has been introduced to overcome the shortcomings of conventional methods. This can be defined as operative procedures that are performed in novel ways to diminish the sequelae of standard surgical dissections [2]. One of the goals of minimally invasive surgery is to minimize bleeding, edema, and injury, thereby improving the rate and quality of healing [3]. Endoscope-assisted surgery is among the minimally invasive approaches used in several medical fields. This method is considered to be a minimally invasive procedure for temporo-mandibular joint surgery, paranasal sinus surgery, salivary duct management, and cosmetic surgery. It not only provides good illumination and clear and magnified visualization of the operative field, but also transfers the image to a monitor. Most of all, the endoscope-assisted technique provides the greatest benefits in terms of minimally invasive approach and reduced morbidity.

The purposes of this article are to describe the early clinical experiences with endoscope-assisted cysts on the jaw enucleation as minimally invasive surgery and to assess if this approach can replace the conventional approach.

## Patients and methods

This was a retrospective case series study of 24 patients (9 females, 15 males, average age 41.5) with bony cysts on the mandible or maxilla. Patients underwent an endoscope-assisted cyst enucleation at Hanyang University Hospital. The diagnosis of jaw cyst was confirmed by clinical examination, panoramic view, and facial CT scan.

**Fig. 1:**
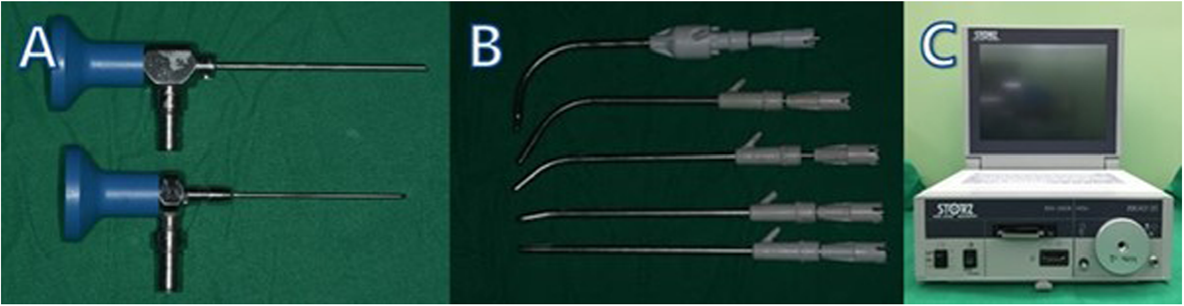
Endoscopic visualization techniques in oral surgery. **A** Support endoscopy. Top: a Stryker endoscope (2.7 mm diameter, 30°). Bottom: a Stryker endoscope (1.9 mm diameter, 0°). **B** Straight and curved sinus blades (4 mm diameter, 90°; 4 mm diameter, 60°; 4 mm diameter, 40°; 4 mm diameter, 12°; 4 mm diameter, 0°; Medtronic). **C** The endoscope mobile unit with endoscope, monitor, light source, and archive system (Karl Storz)

The endoscopic equipment consisted of rigid endoscopes of 2.7 and 1.9 mm diameter with support and irrigation sheaths (Karl Storz®, Tuttlingen, Germany). The endoscopes were linked to a Storz 487-B examination unit and a xenon 300-W light fountain with a 6000-K capacity (Karl Storz®, Tuttlingen, Germany). The larger endoscope had a 30° view angle, and the smaller one, a 0° view angle. For adequate visibility, continuous irrigation using the support sheath and straight and curved sinus blades (Medtronic®, Minneapolis, USA) was used (Fig. 1).

This retrospective study (HYUH 2016-04-013) was approved by the Institutional Review Board (IRB) of Hanyang University Hospital. Data were collected from the hospital and clinical charts, as well as from video recordings of the endoscopic procedures. Data included demographics (gender, age, and date of surgery), clinical, and surgical information (Table 1). The clinical information included the diagnosis, name of the surgical procedure, operation site (involved tooth), postoperative symptoms such as pain or swelling, and complications such as infection, tooth vitality, wound dehiscence, recurrence, inferior alveolar nerve damage, paresthesia, and facial esthetics. The surgical information included the existence of bone perforation at the cyst site, the distance from cystic margin to vertical incision, and the number of teeth undergoing apicoectomy. Follow-ups were done at 5 time points: during the immediate postoperative period and after 1 month, 3 months, 6 months, and 12 months postoperatively.

**Table 1:** Data included epidemiologic (gender, age, and date of surgery), clinical, and surgical information

No.	Age	Sex	Diagnosis	Operation name	Group	Op. site
1	69	M	Radicular cyst	Cyst enucleation and apicoectomy	II	#21, 22
2	72	M	Radicular cyst	Cyst enucleation and apicoectomy	II	#44
3	31	F	Dentigerous cyst	Cyst enucleation and extraction	II	#36, 37, 38
4	32	M	Radicular cyst	Cyst enucleation and apicoectomy	II	#11, 12
5	49	M	Dentigerous cyst	Cyst enucleation and extraction	II	#48
6	70	F	Radicular cyst	Cyst enucleation and apicoectomy	II	#15, 16, 17
7	13	F	Radicular cyst	Cyst enucleation	I	#34
8	33	M	Radicular cyst	Cyst enucleation and apicoectomy	I	#11, 12
9	38	M	Radicular cyst	Cyst enucleation and apicoectomy	I	#36
10	49	M	Radicular cyst	Cyst enucleation and apicoectomy	I	#12, 13
11	28	F	Radicular cyst	Cyst enucleation and apicoectomy	I	#42, 41, 31
12	42	F	Radicular cyst	Cyst enucleation and apicoectomy	I	#47
13	19	F	Radicular cyst	Cyst enucleation	II	#25
14	39	M	Radicular cyst	Cyst enucleation and apicoectomy	II	#21, 22
15	40	M	Radicular cyst	Cyst enucleation and apicoectomy	II	#15
16	22	M	Radicular cyst	Cyst enucleation and apicoectomy	II	#26
17	42	M	Radicular cyst	Cyst enucleation	II	#16
18	52	F	Radicular cyst	Cyst enucleation and apicoectomy	I	#44, 45
19	18	F	Dentigerous cyst	Cyst enucleation and extraction	II	#17, 18
20	35	M	Radicular cyst	Cyst enucleation and apicoectomy	II	#21, 22, 23
21	69	M	Radicular cyst	Cyst enucleation and apicoectomy	II	#11, 21, 22, 23
22	14	F	Dentigerous cyst	Cyst enucleation and extraction	I	#38
23	44	M	Dentigerous cyst	Cyst enucleation and extraction	II	#48
24	63	M	Dentigerous cyst	Cyst enucleation and extraction	I	#48

Group I (intact cortical bone), group II (bone penetration)

## Surgical technique

The endoscopic surgery was performed under general anesthesia. All the patients underwent endoscope-assisted cyst enucleation and excisional biopsy by one surgeon. Each case was classified based on whether bone penetration occurred at the cyst’s lateral bony wall.

As the cyst develops, penetration of the cyst’s outer wall may occur. As such, they are classified during the surgery into two groups based on the formation of a small bony hole. Group 1 confined to the intact bone without reaching the outer wall. In group 2, the development of the cysts reached the outer wall and formed perforations of various sizes on the outer wall. These outer wall perforations resulted in different methods of drilling bony holes during surgery.

**Fig. 2:**
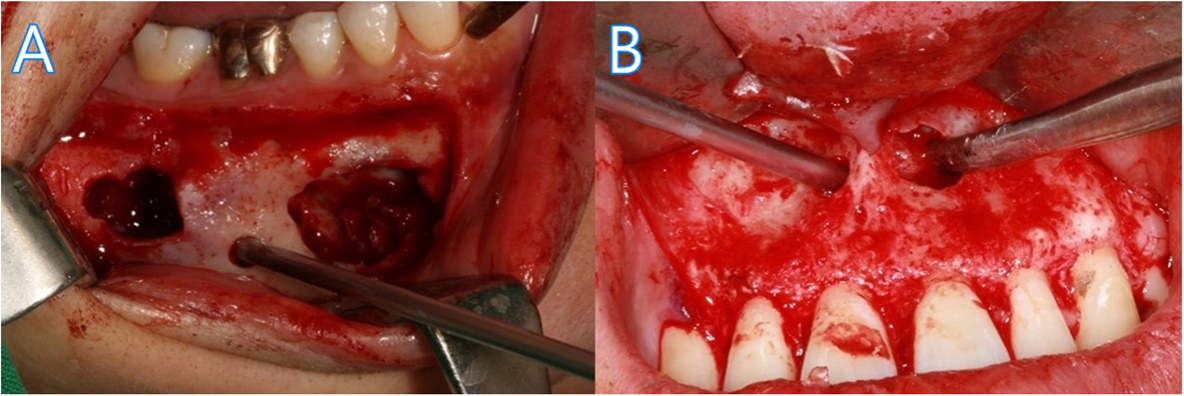
Radicular cyst enucleation with insertion of the endoscope. **A** Group 1 intact cortical bone: made two bony holes for enucleation instrument and additional channel for endoscope. **B** Group 2 cystic bony wall penetrated: penetrated bony wall for enucleation instrument and additional channel was made for endoscope

**Fig. 3:**
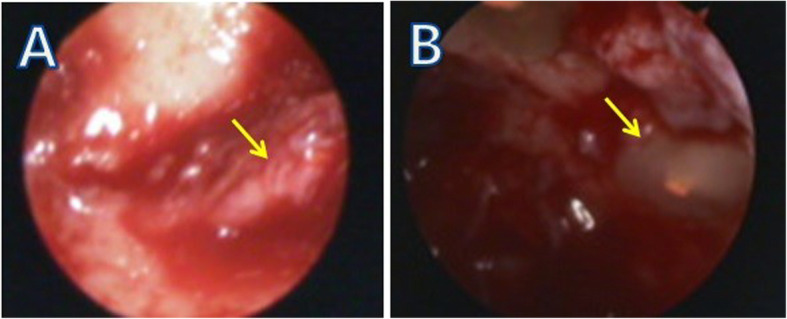
Endoscopic view of the radicular cyst enucleation site. **A** Epithelial remnants. **B** The apicoectomy site of the anterior maxillary tooth

In cases with intact cortical bone (group 1), the muco-gingival flap was elevated to full thickness, and two bony windows were made after examining the cystic bony wall. One window was on one side of the dental arch at one tooth’s width from the cystic margin, and the other was on the opposite side. These two bony windows were used to insert a syringe for irrigation, curettes, suction tips, sinus blades, surgical drills, and the endoscope. In some cases, an additional small channel was made for inserting an endoscopic instrument (Fig. 2). Cyst enucleation, apicoectomy, and excisional biopsy were performed with endoscopic visualization (Fig. 3). The adequate irrigation with isotonic saline was essential for good visibility.

When the cystic bony wall had been penetrated (group 2), the penetrated bony wall was used as the window for inserting the enucleation instrument, and an additional small channel was also made for the endoscope. After the endoscope was inserted, the cyst was enucleated as described above, leading to internal bone healing through internal bone wall space maintenance, such as those used in bone regeneration for implants. Following complete enucleation and excisional biopsy, the cyst cavity was repeatedly irrigated with isotonic saline and antibiotics.

## Results

All patients underwent endoscope-assisted cyst enucleation. The indications for surgery included radicular cyst (*n* = 18) and dentigerous cyst (*n* = 6). There were 13 cysts on the maxilla and 11 on the mandible. The cortical bone was intact in 9 patients (37.5%) and was penetrated in 15 patients (62.5%) (Table 2).

**Table 2:** Patient demographics according to group

	Group 1	Group 2	Summary
Average age (years)	36.9	43.4	41.5
Male (*n* = patients)	4	11	15 (62.5%)
Female (*n* = patients)	5	4	9 (37.5%)
Maxilla (*n* = patients)	2	11	13 (54.2%)
Mandible (*n* = patients)	7	4	11 (45.8%)
Dentigerous cyst (*n* = patients)	2	4	6 (25%)
Radicular cyst (*n* = patients)	7	11	18 (75%)

Group 1 (intact cortical bone), group 2 (bone penetration)

All patients were regularly observed for 3 to 12 months. Eight patients had slight swelling in the operative area, which resolved spontaneously, and the expected esthetic results were obtained. With regard to complications, 2 patients experienced numbness and paresthesia of the lower lip and chin area due to postoperative inferior alveolar nerve damage but soon returned to normal. There was no postoperative blood loss (including oozing) or wound dehiscence. The teeth, which were involved with the cystic lesions, remained vital, and no clinical or radiological evidence of recurrence was observed during follow-ups.

## Discussion

The goals of cystic lesion treatment are to eliminate the possibility of a recurrence and to minimize surgical morbidity. If the lesion is large or difficult to access, the conventional method of cyst enucleation can be invasive. Resnick et al. have reported that minimally invasive approaches in the oral and maxillofacial region may become the standard [4]. These techniques have been shown to reduce bleeding and swelling, minimize scarring, provide superior visualization, create less tension on vital structures, increase stability, and reduce hospital length of stay and recovery time [4]. Huang et al. also reported endoscope surgery reduces blood loss compared to the conventional method [5]. Support of the bone under the flap is required for better soft tissue healing; thus, in conventional methods, large flaps are needed to completely contain cysts; however, by using endoscope, the flap size can be drastically reduced. Also, a small bony hole makes the flaps and bones more stable, which reduces swelling. This can decrease bleeding and postoperative edema. Additionally, in cases requiring extensive incision due to a difficult or extraoral approach, complications, such as damage to the facial nerve and scarring of the skin by the endoscope, can be avoided [6].

Using an endoscope for cyst enucleation is one type of minimally invasive approach. Endoscope-assisted operations have been performed for many years in many fields of general surgery, otolaryngology, obstetrics, gynecology, and so on. Recent advances in imaging, fiber optic technology, and instrumentation may lead to further advancement of endoscopic approach surgery [7]. Kretzschmar et al. reported the intraoral endoscopic enucleation of a solitary mandibular bone cyst, underlining the fact that easy access to the mandibular condyle with good visibility allows for definitive treatment of central lesions of the condyle [8]. Mohamed et al. reported that approaching the maxillary sinus with a sub-labial view by using endoscopic has merits for direct approach. This advantage is not available in conventional nasal approaches due to angulation of nasal passage [9].

With conventional methods, visualization of the operative field is often difficult when approaching the maxillary or mandibular posterior areas. In poor visualization of the operative field, residuals are often left in the teeth area or blind spots. Endoscope-assisted cyst enucleation enables superior visualization of the operative field by magnifying the view through the lens. It provides good illumination, clear visualization of the operation field, and, as a result, more accurate surgery is possible [6, 10]. The scope allows the entire cystic lesion to be seen clearly with angled instruments [11]. Incomplete removal of cystic lesions leads to recurrence [12]. On the other hand, by using the endoscope, it is possible to remove the cystic cavity, eliminating epithelial remnants within the bone or behind the roots of the tooth, using direct vision and curved cutting tools and drills [11, 13]. So, we can reduce the size of the bony hole because we can obtain good visualization of the right down area of the bone wall and increase the possibility of bone formation inside the cyst area by maintaining the space of the outer wall. When the endoscope was used in most of the operations, it was easy to identify the mandibular notch, lingula, mandibular angle, and maxillary sinus, which reduced recurrence. Also, by digitally observing the operation field on a monitor, it was possible to teach the technique and record the operation [14].

Endoscope-assisted surgery requires basic endoscopic equipment and special instruments. The instruments must be modified and minimized for this technique. The endoscope can be mounted on the retractor used to maintain the optical cavity. Also, one can use a drill or irrigation syringe mounted on the endoscope to make a small bony window. The commonly used rigid nasal endoscope is too long and bulky for dento-alveolar surgery and makes it difficult to achieve the necessary field angle during apicoectomy and retrograde filling of a posterior mandibular tooth. In such circumstances, a good surgical view can be obtained using the 30° or 70° angled endoscope. The arthroscope that is used for TMJ surgery is also a suitable size for cyst enucleation and is easy to handle. The visual field obtained with an arthroscope, however, is small, and the illumination is grainy. Beltran et al. classified endoscopes by the size of the surgical field [15]. There are several types of endoscopes: a direct endoscope is a rigid rod lens endoscope that is introduced directly into a cavity to visualize the surgical field. Immersion endoscope is used to perform surgery in cavities that are not accessible with simple inspection due to contamination. When the endoscope is used in a support sheath, the assembly is called support endoscope [16]. They claimed that a support endoscope (SE) allowed visualization of a 4- to 7-mm surgical field while an immersion endoscopy (IE) had a smaller observation distance of 2 to 3 mm under continuous irrigation. The choice of the appropriate endoscope and position for this technique remains a challenge. Therefore, modified, minimal, and easily operable instruments are needed for enucleating jaw cysts [15].

Endoscope-assisted surgery has a problematic aspect where both the microscope and surgical instruments need to pass through a single hole, making it difficult to operate due to limited visibility. This problem can be resolved by using multiport techniques, which creates two holes for the instrument and one hole for the microscope. This not only ensures secure manipulation of the instrument, but also the visibility [17]. Also, surgery time is reduced, and the technique is less precarious than with a single port. Therefore, a non-expert operator can achieve satisfactory results [18]. According to Huang et al., surgery time changes over time based on an operator’s proficiency. Endoscope surgery can be performed faster than traditional methods by skilled technicians, and this dual-port technique facilitates a shorter surgery [5]. The endoscope provides a magnified two-dimensional video image, so the operator needs to be skilled in specific eye-hand coordination [6]. Oral and maxillofacial surgeons who try to perform the endoscopic procedure must receive sufficient training and obtain adequate surgical experience of the relevant anatomy and understanding of the three-dimensional concept. Furthermore, operating team coordination is important in this approach. These factors help to smooth the surgical procedure and reduce operating time.

In conventional methods, a large hole must be created to completely remove the cystic wall. Guanqi et al. have reported that large burr hole defects have a higher tendency to be incompletely healed and prevent bone healing [19]. The number of walls surrounding the bone has a significant impact on bone healing, as adjacent bone walls contain both osteogenic and angiogenic cells [20]. Therefore, if a small bony hole is created, the possibility of bone healing is greater than traditional methods. Fixing a bony window again is the surest method. However, since additional plates and screws, which may need to be removed in the future, would be necessary, it would be best to use a small bony hole instead.

For good visualization of the operation area, continuous irrigation and suction are required during the procedure. Engelke et al. reported that if the endoscope was placed inside an anatomic cavity and supported by any part of the cavity wall under continuous irrigation, good visualization of intraosseous structures was obtained [21]. Nahlieli et al. also demonstrated that endoscopic visualization under continuous irrigation is essential for assessing the anatomic structure in the operative region [22].

## Conclusions

The limitation of this study is that it was designed as a preliminary report focusing on the feasibility of endoscope-assisted cyst enucleation in the oral and maxillofacial region, not as a randomized controlled trial for data analysis in depth. Nevertheless, our results have shown the advantages and effectiveness of minimally invasive surgery with endoscopes for enucleation of jaw cysts.

## Data Availability

The datasets used and/or analyzed during the current study are available from the corresponding author on reasonable request.

## Abbreviation

CT: Computed tomography

## References

[1] Koseoglu BG, Atalay B, Erdem MA (2004). Odontogenic cysts: a clinical study of 90 cases. J Oral Sci.

[2] Hunter JG (1999). Minimally invasive surgery: the next frontier. World J Surg.

[3] Williams WB, Abukawa H, Shuster V, Kaban LB, Troulis MJ (2003) A comparison of postperative edema after introral vs. endoscopic mandibular ramus osteotomy. J Oral Maxillofac Surg 8(61):61a–662a

[4] Resnick CM, Kaban LB, Troulis MJ (2009). Minimally invasive orthognathic surgery. Facial Plast Surg.

[5] Huang Z, Guo W, Zhou B, Chen X (2015). Minimally invasive endoscopic surgery of thyroglossal duct cysts. J Laparoendosc Adv Surg Tech.

[6] Suarez-Cunqueiro MM, Schoen R, Schramm A, Gellrich NC, Schmelzeisen R (2003). Endoscopic approach to removal of an ectopic mandibular third molar. Br J Oral Maxillofac Surg.

[7] Troulis MJ, Perrott DH, Kaban LB (1999). Endoscopic mandibular osteotomy and placement and activation of a semiburied distractor. J Oral Maxillofac Surg.

[8] Kretzschmar DP, Postma GN, Inman JL (2005). Intraoral endoscopic enucleation of a central mandibular condylar lesion. J Oral Maxillofac Surg.

[9] Abdelwahab M, Abd Elfattah AM, Khafagy YW, El-Degwi A (2018). Endoscopic enucleation of large jaw cysts: promising outcomes. Auris Nasus Larynx.

[10] Troulis MJ, Kaban LB (2001). Endoscopic approach to the ramus/condyle unit: clinical applications. J Oral Maxillofac Surg.

[11] Sembronio S, Albiero AM, Zerman N, Costa F, Politi M (2009). Endoscopically assisted enucleation and curettage of large mandibular odontogenic keratocyst. Oral Surg Oral Med Oral Pathol Oral Radiol Endod.

[12] Morgan TA, Burton CC, Qian F (2005). A retrospective review of treatment of the odontogenic keratocyst. J Oral Maxillofac Surg.

[13] Giovannetti F, Cassoni A, Battisti A, Gennaro P, Della Monaca M, Valentini V (2010). Endoscopic approach to benign lesion involving the mandibular condyle. J Craniofac Surg.

[14] Hundepool AC, Willemsen MA, Koudstaal MJ, van der Wal KG (2012). Open reduction versus endoscopically controlled reconstruction of orbital floor fractures: a retrospective analysis. Int J Oral Maxillofac Surg.

[15] Beltran V, Fuentes R, Engelke W (2014). Endoscopic visualization of anatomic structures as a support tool in oral surgery and implantology. J Oral Maxillofac Surg.

[16] Engelke W, Beltrán V (2014). Endoscopic techniques in minimally invasive oral surgery.

[17] Stenin I, Hansen S, Becker M, Sakas G, Fellner D, Klenzner T, Schipper J (2014). Minimally invasive multiport surgery of the lateral skull base. BioMed research international.

[18] Kim SM, Baek JM, Park EK, Jeung IC, Choi JH, Kim CJ, Lee YS (2015). A comparison of single-, two-and three-port laparoscopic myomectomy. JSLS.

[19] Liu G, Guo Y, Zhang L, Wang X, Liu R, Huang P, Xiao Y, Chen Z, Chen Z (2019). A standardized rat burr hole defect model to study maxillofacial bone regeneration. Acta Biomater.

[20] Ettl T, Gosau M, Sader R, Reichert TE (2012). Jaw cysts–filling or no filling after enucleation? A review. J Craniomaxillofac Surg.

[21] Engelke WG (2002). In situ examination of implant sites with support immersion endoscopy. Int J Oral Maxillofac Implants.

[22] Nahlieli O, Moshonov J, Zagury A, Michaeli E, Casap N (2011). Endoscopic approach to dental implantology. J Oral Maxillofac Surg.

